# The genome sequence of a stonefly, *Nemurella pictetii* Klapalek, 1900

**DOI:** 10.12688/wellcomeopenres.17684.1

**Published:** 2022-02-15

**Authors:** Craig Macadam, Caleala Clifford, Benjamin W. Price, Natural History Museum Genome Acquisition Lab, Darwin Tree of Life Barcoding collective, Wellcome Sanger Institute Tree of Life programme, Wellcome Sanger Institute Scientific Operations: DNA Pipelines collective, Tree of Life Core Informatics collective, Darwin Tree of Life Consortium

**Affiliations:** 1 Buglife – The Invertebrate Conservation Trust, Stirling, UK; 2 South East Environmental Assessment and Advice Team, Natural Resources Wales, Cardiff, UK; 3 Department of Life Sciences, Natural History Museum, London, UK

**Keywords:** Nemurella pictetii, genome sequence, chromosomal, Plecoptera

## Abstract

We present a genome assembly from an individual male *Nemurella pictetii* (Arthropoda; Insecta; Plecoptera; Nemouridae). The genome sequence is 257 megabases in span. The majority of the assembly (99.79%) is scaffolded into 12 chromosomal pseudomolecules, with the X sex chromosome assembled. The X chromosome was found at half coverage, but no Y chromosome was found. The mitochondrial genome was assembled, and is 16.0 kb in length.

## Species taxonomy

Eukaryota; Metazoa; Ecdysozoa; Arthropoda; Hexapoda; Insecta; Pterygota; Neoptera; Polyneoptera; Plecoptera; Nemouroidea; Nemouridae; Nemourinae; Nemurella; *Nemurella pictetii* Klapalek, 1900 (NCBI:txid143722).

## Background

*Nemurella pictetii* is a western palearctic species found from northern Spain to Siberia, and throughout the British Isles, although appears to be less common in Scotland. It is considered a eurytherm and can be found in a variety of habitats from rivers and streams to ponds and lakes. It does not appear to have any altitudinal preference; however, in Ireland it is more usually encountered in streams draining peaty soils usually at higher altitudes and in small headwater streams and seepages ( Baars & Kelly-Quinn, 2006; Hynes, 1977). In Great Britain and Europe, larvae are known to occur in small upland lakes with stony shorelines and in vegetated ponds, wetlands and marshes (e.g. ( Lillehammer, 1975; Macadam, 2015; Wolf & Zwick, 1989)). Larvae are often common and can be numerous in small trickles and streams with dense vegetation, woody material and/or organic matter ( Costello *et al*., 1984; Feeley *et al*., 2019; Graf *et al*., 2009; Wolf & Zwick, 1989), but generally they are scarce, only occurring in low densities. They are also highly tolerant of low pH and conductivity ( Baars & Kelly-Quinn, 2006; Feeley, 2012; Murphy *et al*., 2013). Both ( Brittain, 1991) and ( Baars & Kelly-Quinn, 2006) report this species as somewhat tolerant of nutrient enrichment. Larvae are opportunistic feeders utilising a broad range of food sources, but preferring biofilm ( Lieske & Zwick, 2007).

*Nemurella pictetii* is the sole representative of the genus *Nemurella*. The high-quality genome sequence described here is, to our knowledge, the first one reported for *N. pictetii*, and has been generated as part of the Darwin Tree of Life project. It will aid in understanding the biology, physiology and ecology of the species. 

## Genome sequence report

**Figure 1.**
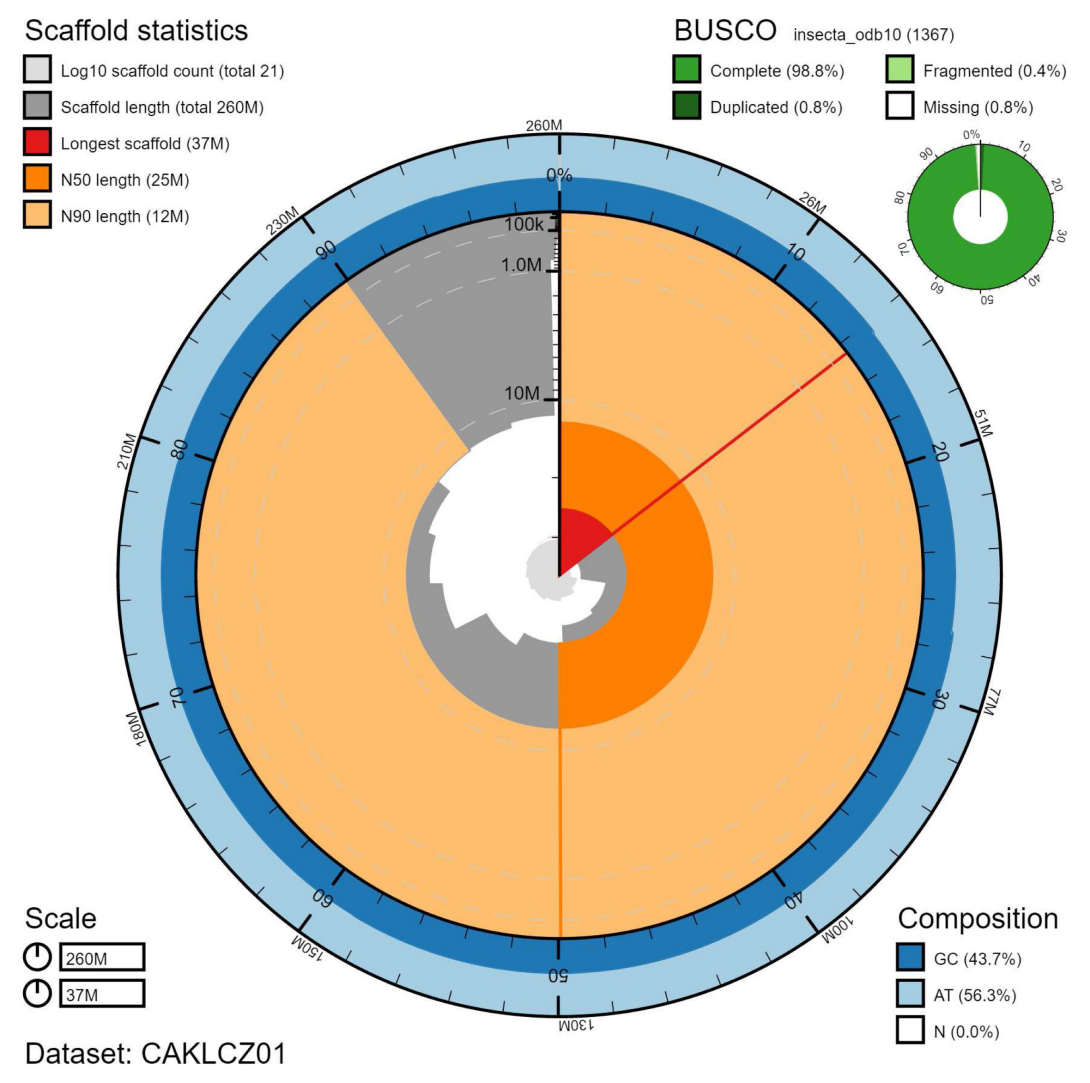
Genome assembly of *Nemurella pictetii*, ipNemPict2.1: metrics. The BlobToolKit Snailplot shows N50 metrics and BUSCO gene completeness. The main plot is divided into 1,000 size-ordered bins around the circumference with each bin representing 0.1% of the 257,052,056 bp assembly. The distribution of scaffold lengths is shown in dark grey with the plot radius scaled to the longest scaffold present in the assembly (37,341,160 bp, shown in red). Orange and pale-orange arcs show the N50 and N90 scaffold lengths (24,564,665 and 12,448,030 bp), respectively. The pale grey spiral shows the cumulative scaffold count on a log scale with white scale lines showing successive orders of magnitude. The blue and pale-blue area around the outside of the plot shows the distribution of GC, AT and N percentages in the same bins as the inner plot. A summary of complete, fragmented, duplicated and missing BUSCO genes in the insecta_odb10 set is shown in the top right. An interactive version of this figure is available at https://blobtoolkit.genomehubs.org/view/CAKLCZ01/dataset/CAKLCZ01/snail.

The genome was sequenced from one female *N. pictetii* ( Figure 1) collected from River Taff Fawr, Garwnant, Wales (latitude 51.8082, longitude -3.4449). A total of 68-fold coverage in Pacific Biosciences single-molecule long reads and 183-fold coverage in 10X Genomics read clouds were generated. Primary assembly contigs were scaffolded with chromosome conformation Hi-C data. Manual assembly curation corrected 62 missing/misjoins and removed 8 haplotypic duplications, reducing the assembly size by 2.35% and scaffold number by 65.52% and increasing the scaffold N50 by 8.35%.

The final assembly has a total length of 257 Mb in 20 sequence scaffolds with a scaffold N50 of 24.6 Mb ( Table 1). The majority of the assembly sequence (99.79%) was assigned to 12 chromosomal-level scaffolds, representing 11 autosomes (numbered by sequence length), and the X sex chromosome ( Figure 1– Figure 4; Table 2). The sex of the specimen was determined to be male as chromosome X was found at half coverage, despite no evidence of the presence of a Y chromosome. The assembly has a BUSCO v5.1.2 ( Manni *et al*., 2021) completeness of 98.8% (single 98.0%, duplicated 0.8%) using the insecta_odb10 reference set (n=1,367). While not fully phased, the assembly deposited is of one haplotype. Contigs corresponding to the second haplotype have also been deposited.

**Table 1.** Genome data for *Nemurella pictetii*, ipNemPict2.1.

*Project accession data*	
Assembly identifier	ipNemPict2.1
Species	*Nemurella pictetii*
Specimen	ipNemPict2 (male, genome assembly); ipNemPict1 (unknown sex, Hi-C)
NCBI taxonomy ID	NCBI:txid143722
BioProject	PRJEB46304
BioSample ID	SAMEA7521125
Isolate information	Male, whole organism (ipNemPict2); unknown sex, whole organism (ipNemPict1)
*Raw data accessions*	
PacificBiosciences SEQUEL II	ERR6808073, ERR6909090, ERR6939286, ERR6939287
10X Genomics Illumina	ERR6688441-ERR6688448
Hi-C Illumina	ERR6688440
*Genome assembly*	
Assembly accession	GCA_921293315.1
Accession of alternate haplotype	GCA_921293065.1
Span (Mb)	257
Number of contigs	105
Contig N50 length (Mb)	9.6
Number of scaffolds	20
Scaffold N50 length (Mb)	24.6
Longest scaffold (Mb)	37.3
BUSCO * genome score	C:98.8%[S:98.0%,D:0.8%], F:0.4%,M:0.8%,n:1367

*BUSCO scores based on the insecta_odb10 BUSCO set using v5.1.2. C= complete [S= single copy, D=duplicated], F=fragmented, M=missing, n=number of orthologues in comparison. A full set of BUSCO scores is available at https://blobtoolkit.genomehubs.org/view/ipNemPict2.1/dataset/CAKLCZ01/busco.

**Figure 2.**
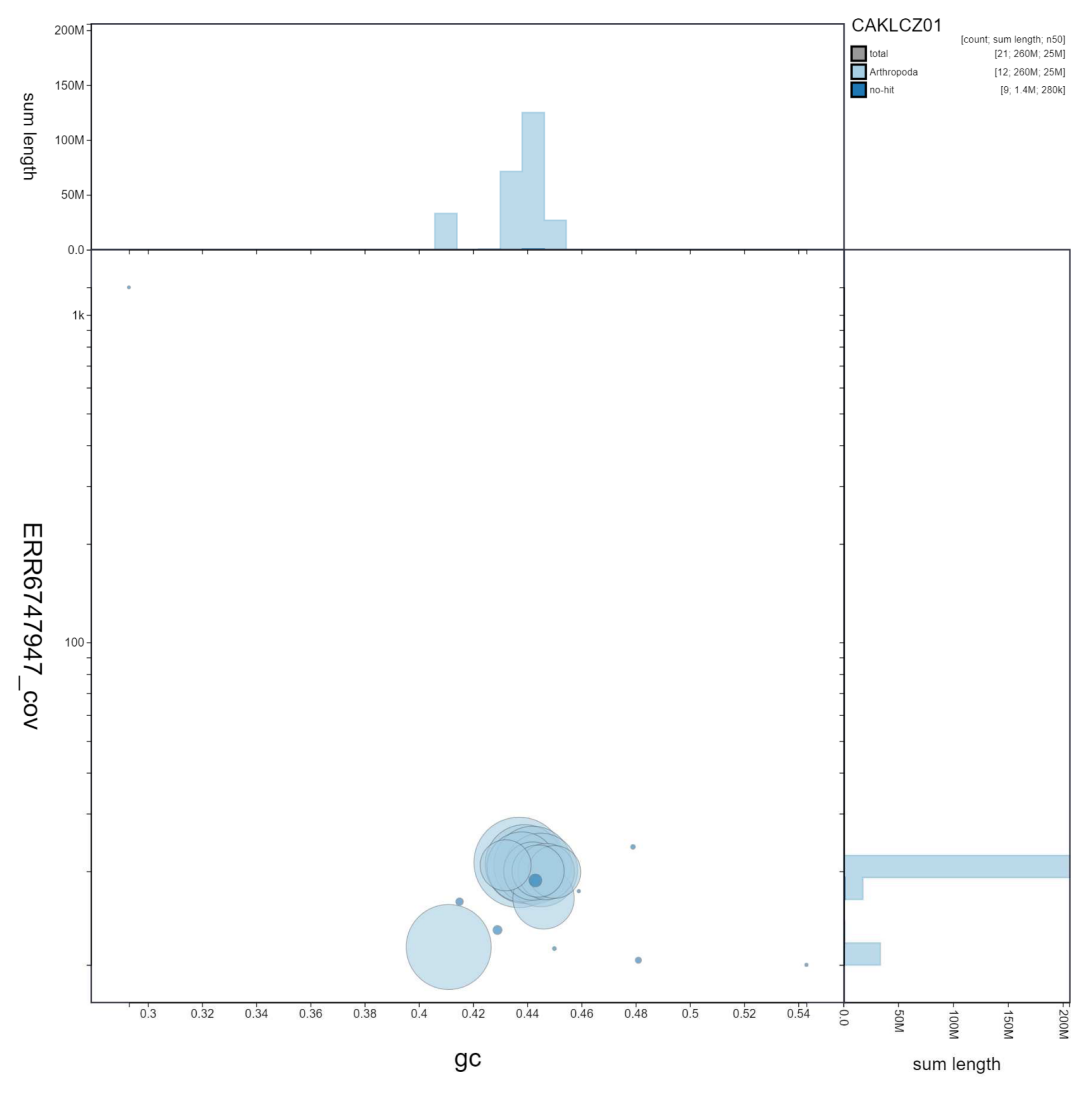
Genome assembly of *Nemurella pictetii*, ipNemPict2.1: GC coverage. BlobToolKit GC-coverage plot. Scaffolds are coloured by phylum. Circles are sized in proportion to scaffold length Histograms show the distribution of scaffold length sum along each axis. An interactive version of this figure is available at https://blobtoolkit.genomehubs.org/view/CAKLCZ01/dataset/CAKLCZ01/blob.

**Figure 3.**
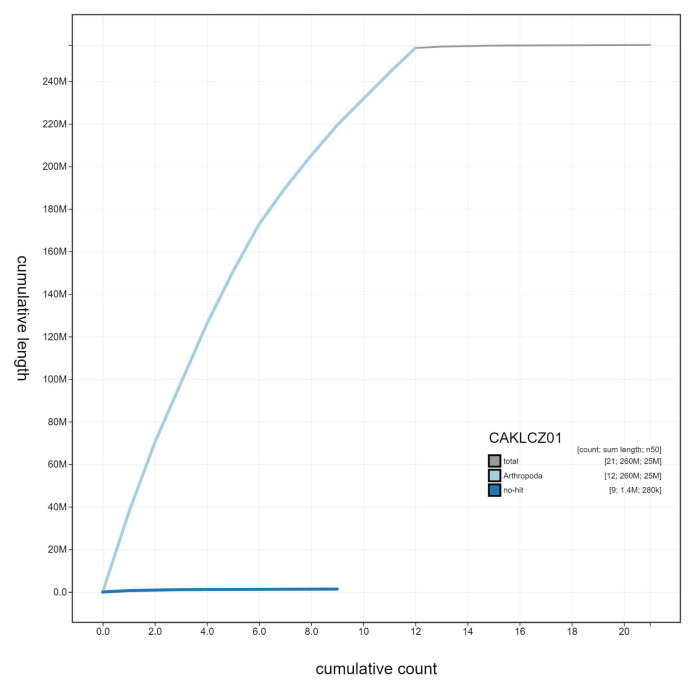
Genome assembly of *Nemurella pictetii*, ipNemPict2.1: cumulative sequence. BlobToolKit cumulative sequence plot. The grey line shows cumulative length for all scaffolds. Coloured lines show cumulative lengths of scaffolds assigned to each phylum using the buscogenes taxrule. An interactive version of this figure is available at https://blobtoolkit.genomehubs.org/view/CAKLCZ01/dataset/CAKLCZ01/cumulative.

**Figure 4.**
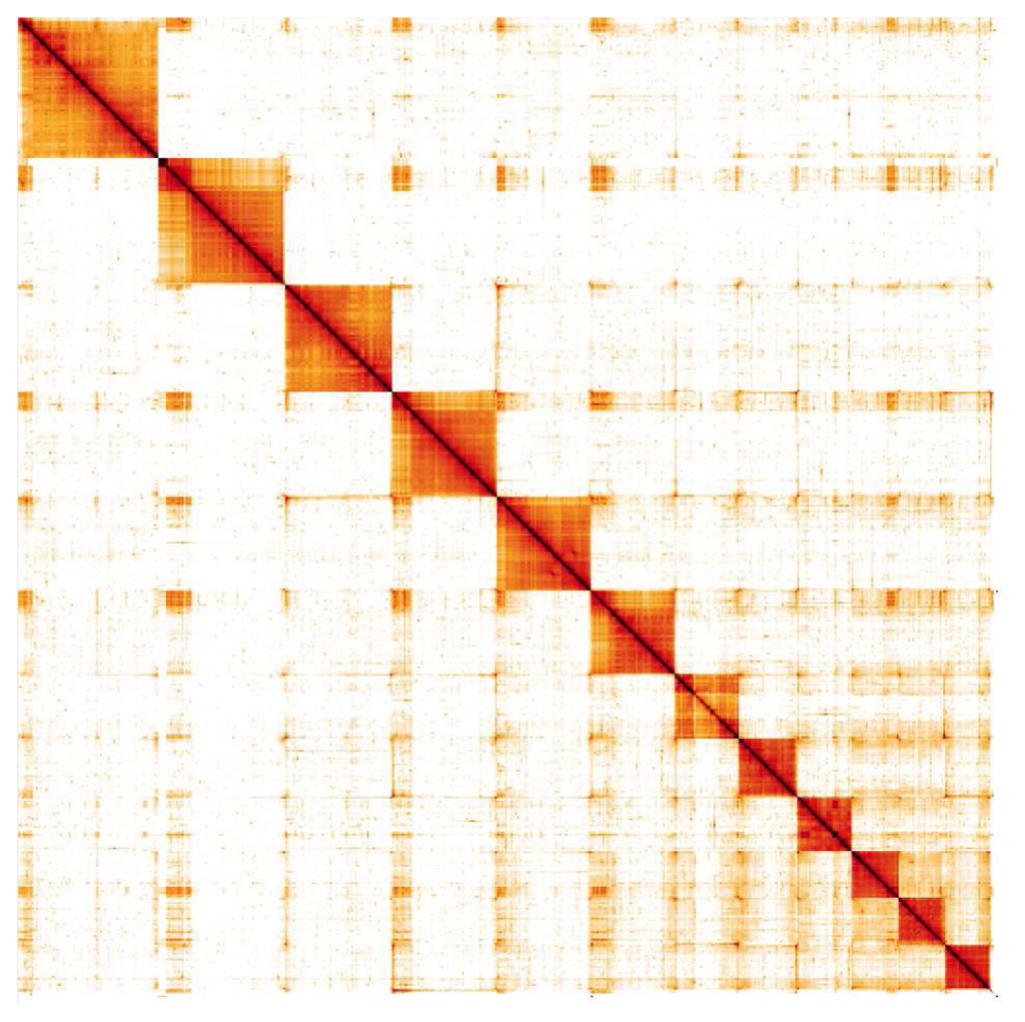
Genome assembly of *Nemurella pictetii*, ipNemPict2.1: Hi-C contact map. Hi-C contact map of the ipNemPict2.1 assembly, visualised in HiGlass. Chromosomes are shown in order of size from left to right and top to bottom.

**Table 2.** Chromosomal pseudomolecules in the genome assembly of *Nemurella pictetii*, ipNemPict2.1.

INSDC accession	Chromosome	Size (Mb)	GC%
OV121115.1	1	37.34	43.7
OV121117.1	2	28.23	43.9
OV121118.1	3	27.56	44.2
OV121119.1	4	24.56	44.5
OV121120.1	5	22.19	43.8
OV121121.1	6	17.00	44.6
OV121122.1	7	15.30	44.2
OV121123.1	8	14.27	44.7
OV121124.1	9	12.45	45.0
OV121125.1	10	12.20	44.4
OV121126.1	11	11.63	43.2
OV121116.1	X	32.95	41.1
OV121127.1	MT	0.02	29.3
-	Unplaced	1.35	44.3

## Methods

### Sample acquisition and DNA extraction

Two *N. pictetii* specimens (ipNemPict1, unknown sex, and ipNemPict2, male) were collected from River Taff Fawr, Garwnant, Wales (latitude 51.8082, longitude -3.4449) by Natural Resources Wales using a kick-net. The sample was identified by representatives of the same body and snap-frozen in liquid nitrogen. Unfortunately, no images of the samples are available.

DNA was extracted at the Tree of Life laboratory, Wellcome Sanger Institute. The ipNemPict2 sample was weighed and dissected on dry ice. Whole organism tissue was cryogenically disrupted to a fine powder using a Covaris cryoPREP Automated Dry Pulveriser, receiving multiple impacts. Fragment size analysis of 0.01–0.5 ng of DNA was then performed using an Agilent FemtoPulse. High molecular weight (HMW) DNA was extracted using the Qiagen MagAttract HMW DNA extraction kit. Low molecular weight DNA was removed from a 200-ng aliquot of extracted DNA using 0.8X AMpure XP purification kit prior to 10X Chromium sequencing; a minimum of 50 ng DNA was submitted for 10X sequencing. HMW DNA was sheared into an average fragment size between 12–20 kb in a Megaruptor 3 system with speed setting 30. Sheared DNA was purified by solid-phase reversible immobilisation using AMPure PB beads with a 1.8X ratio of beads to sample to remove the shorter fragments and concentrate the DNA sample. The concentration of the sheared and purified DNA was assessed using a Nanodrop spectrophotometer and Qubit Fluorometer and Qubit dsDNA High Sensitivity Assay kit. Fragment size distribution was evaluated by running the sample on the FemtoPulse system.

### Sequencing

Pacific Biosciences HiFi circular consensus and 10X Genomics Chromium read cloud sequencing libraries were constructed according to the manufacturers’ instructions. Sequencing was performed by the Scientific Operations core at the Wellcome Sanger Institute on Pacific Biosciences SEQUEL II (HiFi) and Illumina NovaSeq 6000 (10X) instruments. Hi-C data were generated from whole organism tissue of ipNemPict1 using the Arima Hi-C+ kit and sequenced on an Illumina HiSeq X instrument.

### Genome assembly

Assembly was carried out with Hifiasm ( Cheng *et al*., 2021); haplotypic duplication was identified and removed with purge_dups ( Guan *et al*., 2020). One round of polishing was performed by aligning 10X Genomics read data to the assembly with longranger align, calling variants with freebayes ( Garrison & Marth, 2012). The assembly was then scaffolded with Hi-C data ( Rao *et al*., 2014) using SALSA2 ( Ghurye *et al*., 2019). The assembly was checked for contamination as described previously ( Howe *et al*., 2021). Manual curation ( Howe *et al*., 2021) was performed using HiGlass ( Kerpedjiev *et al*., 2018) and Pretext. The mitochondrial genome was assembled using MitoHiFi ( Uliano-Silva *et al*., 2021), which performs annotation using MitoFinder ( Allio *et al*., 2020). The genome was analysed and BUSCO scores generated within the BlobToolKit environment ( Challis *et al*., 2020). Table 3 contains a list of all software tool versions used, where appropriate.

**Table 3.** Software tools used.

Software tool	Version	Source
Hifiasm	0.15.3	( Cheng *et al*., 2021)
purge_dups	1.2.3	Guan *et al*., 2020
SALSA2	2.2	Ghurye *et al*., 2019
longranger align	2.2.2	https://support.10xgenomics.com/genome-exome/ software/pipelines/latest/advanced/other-pipelines
freebayes	1.3.1-17-gaa2ace8	Garrison & Marth, 2012
MitoHiFi	2.0	( Uliano-Silva *et al*., 2021)
HiGlass	1.11.6	( Kerpedjiev *et al*., 2018)
PretextView	0.2.x	https://github.com/wtsi-hpag/PretextView
BlobToolKit	2.6.4	Challis *et al*., 2020

### Ethics/compliance issues

The materials that have contributed to this genome note have been supplied by a Darwin Tree of Life Partner. The submission of materials by a Darwin Tree of Life Partner is subject to the Darwin Tree of Life Project Sampling Code of Practice. By agreeing with and signing up to the Sampling Code of Practice, the Darwin Tree of Life Partner agrees they will meet the legal and ethical requirements and standards set out within this document in respect of all samples acquired for, and supplied to, the Darwin Tree of Life Project. Each transfer of samples is further undertaken according to a Research Collaboration Agreement or Material Transfer Agreement entered into by the Darwin Tree of Life Partner, Genome Research Limited (operating as the Wellcome Sanger Institute), and in some circumstances other Darwin Tree of Life collaborators.

## Data availability

European Nucleotide Archive: Nemoura pictetii. Accession number PRJEB47468; https://identifiers.org/ena.embl/PRJEB47468 [identifiers.org].

The genome sequence is released openly for reuse. The *N. pictetii* genome sequencing initiative is part of the Darwin Tree of Life (DToL) project. All raw sequence data and the assembly have been deposited in INSDC databases. The genome will be annotated and presented through the Ensembl pipeline at the European Bioinformatics Institute. Raw data and assembly accession identifiers are reported in Table 1.

## References

[ref-1] Allio R Schomaker-Bastos A Romiguier J : MitoFinder: Efficient Automated Large-Scale Extraction of Mitogenomic Data in Target Enrichment Phylogenomics. *Mol Ecol Resour.* 2020;20(4):892–905. 10.1111/1755-0998.13160 32243090 PMC7497042

[ref-2] Baars JR Kelly-Quinn M : The Plecoptera of Irish Freshwaters--Species Distribution, Status and Association with Environmental Parameters. Report to The Heritage Council of Ireland, Wildlife Grant Scheme.2006;1–26.

[ref-3] Brittain JE : Life History Characteristics as a Determinant of the Response of Mayflies and Stoneflies to Man-Made Environmental Disturbance (Ephemeroptera and Plecoptera). *Overview and Strategies of Ephemeroptera and Plecoptera.*Sandhill Crane Press. Gainesville, Fla. USA.1991. Reference Source

[ref-4] Challis R Richards E Rajan J : BlobToolKit - Interactive Quality Assessment of Genome Assemblies. *G3 (Bethesda).* 2020;10(4):1361–74. 10.1534/g3.119.400908 32071071 PMC7144090

[ref-5] Cheng H Concepcion GT Feng X : Haplotype-Resolved *de Novo* Assembly Using Phased Assembly Graphs with Hifiasm. *Nat Methods.* 2021;18(2):170–75. 10.1038/s41592-020-01056-5 33526886 PMC7961889

[ref-6] Costello MJ McCarthy TK O’Farrell MM : The Stoneflies (Plecoptera) of the Corrib Catchment Area, Ireland. *Annls Limnol.* 1984;20(1–2):25–34. 10.1051/limn/1984014

[ref-7] Feeley HB Little R Baars JR : Some Additional Notes on the Life Histories of Nemurella Pictetii Klapálek and Brachyptera Risi (Morton) (Plecoptera) in Ireland. *Ir Nat J.* 2019;36(2):146–147. Reference Source

[ref-8] Feeley HB : The Impact of Mature Conifer Forest Plantations on the Hydrochemical and Ecological Quality of Headwater Streams in Ireland, with Particular Reference to Episodic Acidification. University College Dublin.2012.

[ref-9] Garrison E Marth G : Haplotype-Based Variant Detection from Short-Read Sequencing. arXiv: 1207.3907.2012. Reference Source

[ref-10] Ghurye J Rhie A Walenz BP : Integrating Hi-C Links with Assembly Graphs for Chromosome-Scale Assembly. *PLoS Comput Biol.* 2019;15(8):e1007273. 10.1371/journal.pcbi.1007273 31433799 PMC6719893

[ref-11] Graf W Lorenz AW de Figueroa JMT : Plecoptera. Coronet Books Incorporated.2009. Reference Source

[ref-12] Guan D McCarthy SA Wood J : Identifying and Removing Haplotypic Duplication in Primary Genome Assemblies. *Bioinformatics.* 2020;36(9):2896–2898. 10.1093/bioinformatics/btaa025 31971576 PMC7203741

[ref-13] Howe K Chow W Collins J : Significantly Improving the Quality of Genome Assemblies through Curation. *GigaScience.* 2021;10(1):giaa153. 10.1093/gigascience/giaa153 33420778 PMC7794651

[ref-14] Hynes HBN : A Key to the Adults and Nymphs of the British Stoneflies (Plecoptera): With Notes on Their Ecology and Distribution. Freshwater Biological Association.1977;17. Reference Source

[ref-15] Kerpedjiev P Abdennur N Lekschas F : HiGlass: Web-Based Visual Exploration and Analysis of Genome Interaction Maps. *Genome Biol.* 2018;19(1):125. 10.1186/s13059-018-1486-1 30143029 PMC6109259

[ref-16] Lieske R Zwick P : Food Preference, Growth and Maturation of *Nemurella Pictetii* (Plecoptera: Nemouridae). *Freshw Biol.* 2007;52(7):1187–97. 10.1111/j.1365-2427.2007.01755.x

[ref-17] Lillehammer A : Norwegian Stoneflies. III. Field Studies on Ecological Factors Influencing Distribution. *Nor J Entomol.* 1975;22:71–80.

[ref-18] Macadam CR : A Review of the Stoneflies (Plecoptera) of Great Britain: Species Status No.20. Commissioned Report NECR174. Natural England.2015. Reference Source

[ref-19] Manni M Berkeley MR Seppey M : BUSCO Update: Novel and Streamlined Workflows along with Broader and Deeper Phylogenetic Coverage for Scoring of Eukaryotic, Prokaryotic, and Viral Genomes. *Mol Biol Evol.* 2021;38(10):4647–54. 10.1093/molbev/msab199 34320186 PMC8476166

[ref-20] Murphy JF Davy-Bowker J McFarland B : A Diagnostic Biotic Index for Assessing Acidity in Sensitive Streams in Britain. *Ecol Indic.* 2013;24:562–72. 10.1016/j.ecolind.2012.08.014

[ref-21] Rao SS Huntley MH Durand NC : A 3D Map of the Human Genome at Kilobase Resolution Reveals Principles of Chromatin Looping. *Cell.* 2014;159(7):1665–80. 10.1016/j.cell.2014.11.021 25497547 PMC5635824

[ref-22] Uliano-Silva M Nunes JGF Krasheninnikova K : marcelauliano/MitoHiFi: mitohifi_v2.0.2021. 10.5281/zenodo.5205678

[ref-23] Wolf B Zwick P : Plurimodal Emergence and Plurivoltinism of Central European Populations of *Nemurella Pictetii* (Plecoptera: Nemouridae). *Oecologia.* 1989;79(4):431–38. 10.1007/BF00378657 28313474

