# Supplementary material for: The genome sequence of a stonefly, Nemurella pictetii Klapalek, 1900
Source: Wellcome Open Res. 2022 Feb 25;7:56. Originally published 2022 Feb 15. [Version 2] doi: 10.12688/wellcomeopenres.17684.2 (PMC9086528; doi:10.12688/wellcomeopenres.17684.2)
Supplement: Peer review history

# Peer review: Reviewer response for version 2

**DOI:** 10.21956/wellcomeopenres.19633.r49820

**Published:** 2022-05-12

**Authors:** Andrew J. Veale

**Affiliations:** 1 Manaaki Whenua Landcare Research, Lincoln, New Zealand

In the Background section, "although appears to be less common in Scotland" should be "although** ***it*** **appears to be less common in Scotland".

 In the methods you should identify what life stage each of the individuals was - are these adults or juveniles, and if juvenile, which instar?  

 In stonefly genomics I have found issues with contamination from filamentous algae and diatoms in particular, which you appear not to have had issues with. When you say whole organism tissue what do you mean? Was the gut present? Was the outer cuticle present? My assumption is that you had an adult and dissected out the gut but it would be good to confirm.

 Were any tissues stored for museum accession?

 Was species ID confirmed through mitochondrial sequences?  

 Having an XO sex determination system appears relatively common in Plecoptera, as it appears to be in this species ^1^. This should probably be made explicit.

 I have no issues with the genome assembly and methods therein - it looks like a high quality assembly.

 Good luck!

Are sufficient details of methods and materials provided to allow replication by others?

Partly

Is the rationale for creating the dataset(s) clearly described?

Yes

Are the datasets clearly presented in a useable and accessible format?

Yes

Are the protocols appropriate and is the work technically sound?

Yes

Reviewer Expertise:

Genomics - have previously worked on Plecoptera genomics.

I confirm that I have read this submission and believe that I have an appropriate level of expertise to confirm that it is of an acceptable scientific standard, however I have significant reservations, as outlined above.

## References

[1] : Sex Determination, Sex Chromosomes, and Karyotype Evolution in Insects. *J Hered* .108(1) : 10.1093/jhered/esw047 78-93 10.1093/jhered/esw047 27543823PMC6281344

# Peer review: Reviewer response for version 2

**DOI:** 10.21956/wellcomeopenres.19633.r50257

**Published:** 2022-05-09

**Authors:** Joshua Benoit

**Affiliations:** 1 Department of Biological Sciences, University of Cincinnati, Cincinnati, OH, USA

This study provides the details on the genome sequence of a stonefly, *Nemurella pictetii. *No additional comments as issues found by the other reviewer have been addressed. Sufficient background and information is provided for this genome. The genome is high quality and will provide a new resource for an understudied system.

Are sufficient details of methods and materials provided to allow replication by others?

Yes

Is the rationale for creating the dataset(s) clearly described?

Yes

Are the datasets clearly presented in a useable and accessible format?

Yes

Are the protocols appropriate and is the work technically sound?

Yes

Reviewer Expertise:

Arthropod biology

I confirm that I have read this submission and believe that I have an appropriate level of expertise to confirm that it is of an acceptable scientific standard.

# Peer review: Reviewer response for version 2

**DOI:** 10.21956/wellcomeopenres.19633.r48912

**Published:** 2022-03-02

**Authors:** Scott Hotaling

**Affiliations:** 1 School of Biological Sciences, Washington State University, Pullman, WA, USA

Approved.

Are sufficient details of methods and materials provided to allow replication by others?

Yes

Is the rationale for creating the dataset(s) clearly described?

Yes

Are the datasets clearly presented in a useable and accessible format?

Yes

Are the protocols appropriate and is the work technically sound?

Yes

Reviewer Expertise:

Insect genomics, genome biology, aquatic ecology

I confirm that I have read this submission and believe that I have an appropriate level of expertise to confirm that it is of an acceptable scientific standard.

# Peer review: Reviewer response for version 1

**DOI:** 10.21956/wellcomeopenres.19566.r48724

**Published:** 2022-02-18

**Authors:** Scott Hotaling

**Affiliations:** 1 School of Biological Sciences, Washington State University, Pullman, WA, USA

In this study, Macadam and colleagues report the assembly of a high-quality stonefly genome - the best to date for this entire order of insects. The study and methods contain no notable flaws and I see no issues in terms of its scientific robustness.

**Background:**

- One missing component - placing this genome assembly in context for the group and insects as a whole. It fills a major niche for Plecoptera and adds a key species for aquatic insects broadly which are dramatically underrepresented (see my article Hotaling *et al.* 2020 ^1^). These points can be highlighted for the reader. It might also be useful to note that the three previous stonefly genomes are exceptionally poor-quality and represent perhaps the lowest quality group of assemblies for any insect order (see my article Hotaling *et al*. 2021, Figure 1d ^2^). Adding these references is far from critical to the report's effectiveness. They would simply be "value added" for a future reader seeking broader context in terms of insect genomics.
- Also there are double parentheses in the first paragraph of the section in the PDF, mid-way through. These may need correction.

**Genome sequence report:**

- Described as being a female specimen in the first sentence but later as male. This should be confirmed/corrected.

**Genome assembly:**

- There is a space missing in the PDF between “Table 3” and “contains” on the top of pg. 7.
- In this study, Macadam and colleagues report the assembly of a high-quality stonefly genome—the best to date for this entire order of insects.

Are sufficient details of methods and materials provided to allow replication by others?

Yes

Is the rationale for creating the dataset(s) clearly described?

Yes

Are the datasets clearly presented in a useable and accessible format?

Yes

Are the protocols appropriate and is the work technically sound?

Yes

Reviewer Expertise:

Insect genomics, genome biology, aquatic ecology

I confirm that I have read this submission and believe that I have an appropriate level of expertise to confirm that it is of an acceptable scientific standard.

## References

[1] : Aquatic Insects Are Dramatically Underrepresented in Genomic Research. *Insects* .2020;11(9) : 10.3390/insects11090601 10.3390/insects11090601 32899516PMC7563230

[2] : Long Reads Are Revolutionizing 20 Years of Insect Genome Sequencing. *Genome Biology and Evolution* .2021;13(8) : 10.1093/gbe/evab138 10.1093/gbe/evab138 PMC835821734152413
